# Evaluating the reach, effectiveness, adoption, implementation and maintenance of the Resistance Training for Teens program

**DOI:** 10.1186/s12966-021-01195-8

**Published:** 2021-09-08

**Authors:** Sarah G. Kennedy, Jordan J. Smith, Paul A. Estabrooks, Nicole Nathan, Mike Noetel, Philip J. Morgan, Jo Salmon, Gessika C. Dos Santos, David R. Lubans

**Affiliations:** 1 Priority Research Centre for Physical Activity and Nutrition, School of Education, University of Newcastle, Callaghan, NSW Australia; 2 Department of Health Promotion, University of Nebraska Medical Center, Omaha, NE USA; 3 National Centre of Implementation Science, University of Newcastle, Callaghan, NSW Australia; 4 Hunter New England Population Health, Hunter New England Area Health Service, Newcastle, NSW Australia; 5 College of Health, Medicine and Wellbeing, The University of Newcastle, Newcastle, NSW Australia; 6 Hunter Medical Research Institute, New Lambton Heights, NSW Australia; 7 Institute for Positive Psychology and Education, Australian Catholic University, Sydney, NSW Australia; 8 Institute for Physical Activity and Nutrition (IPAN), Deakin University, Geelong, Australia; 9 Post-Graduate Program in Physical Education Associate UEM/UEM, State University of Londrina, Londrina, Brazil

**Keywords:** School-based, Muscular fitness, Physical activity, Dissemination, Translation

## Abstract

**Background:**

Physical activity guidelines recommend young people engage in regular muscle-strengthening activities (e.g., resistance training [RT]). However, few school-based physical activity interventions have been delivered at-scale or promoted RT. The aim of this study was to evaluate the reach, effectiveness, adoption, implementation and maintenance of the *Resistance Training for Teens* (*RT for Teens*) program.

**Methods:**

Data were collected between August 2015 and October 2020. RE-AIM was operationalized as: (i) *Reach*: number and characteristics of students estimated to be exposed to the program; (ii) *Effectiveness:* impact of the program on student-level outcomes measured in a subsample of 750 students from 17 schools; (iii) *Adoption:* number and representativeness of schools with one or more teachers trained to deliver the program; (iv) *Implementation:* extent to which the program was delivered as intended; and (v) *Maintenance:* extent to which the program was sustained in schools.

**Results:**

The estimated program reach was ~ 10,000 students, out of a total student population of ~ 200,000 (~ 5%). Students were from diverse socioeconomic and ethnic backgrounds. Improvements in muscular fitness, RT self-efficacy, perceived cardiorespiratory fitness and flexibility, and participation in muscle-strengthening physical activities were documented. A total of 30 workshops were delivered, involving 468 teachers from 249 schools from diverse geographical regions. Implementation varied considerably, with teachers adapting the program to suit the context of their school and student cohorts. However, RT skill development and the promotion of muscular fitness were the session components delivered most during sessions. Teachers’ adherence to the SAAFE (Supportive, Active, Autonomous, Fair and Enjoyable) teaching principles was high. Approximately 30% of teachers (144/476) registered to use the RT for Teens app. At the school-level, 37% (93/249) of schools had at least one registered user (teacher and/or student). A total of 2,336 workouts and 3,116 fitness tests were completed by registered users. Of the 249 schools represented, 51 (20.5%) sent an additional (previously untrained) teacher to a second workshop.

**Conclusions:**

The *RT for Teens* program had broad reach and adoption. However, intervention delivery varied considerably across schools and additional support strategies are required to optimize intervention implementation and maintain program delivery over time. Future studies will benefit from the utilization of accepted frameworks, recommendations and guidelines for implementation research.

**Trial registration:**

Australian New Zealand Clinical Trials Registry (ACTRN12621000352808), retrospectively registered 1^st^ February 2021.

**Supplementary Information:**

The online version contains supplementary material available at 10.1186/s12966-021-01195-8.

## Background

The health benefits of muscular fitness for adults [1] and youth [2] are compelling. As a result, international guidelines recommend young people (5–17 years) engage in muscle-strengthening activities (e.g., resistance training [RT]) on at least three days per week [3]. Despite this recommendation, 51% of United States (US) high school students [4], and only 13% of Australians aged 15–17 [5] are meeting this guideline. Adult data from the US [6], Europe [7], and Australia [8] suggest slightly lower participation, with 21.9%, 17.3% and 9.3% meeting muscle-strengthening activity guidelines (i.e., ≥ 2 days/week), respectively [9–11]. A number of barriers to participation in RT exist amongst adults, including low self-efficacy, negative affective judgement (i.e., negative feelings about RT) and lack of self-regulation strategies (e.g., goal setting and self-monitoring for RT) [12]. These barriers are also relevant for adolescents, as they may lack the knowledge, skills and/or confidence to participate, despite their desire to try varied physical activities [13]. In addition to individual-level barriers, parental attitudes towards RT [14] may influence adolescents’ participation opportunities in RT. Providing adolescents with opportunities to develop the necessary skills, knowledge, and self-efficacy [15] to engage in RT may help to overcome these barriers [12] and support lifelong participation.

Secondary schools are well-positioned to introduce young people to RT as they have the access and curriculum to target health behaviors. They are also uniquely placed with personnel, such as physical education (PE) teachers, who may have the expertise to deliver RT programs. Unfortunately, there are notable barriers to the delivery of RT in schools, including lack of appropriate facilities, equipment, teacher confidence, and time [16, 17]. Additionally, teachers may be uncertain of how to integrate RT into school programs, and may subscribe to misconceptions related to the appropriateness of RT for youth (i.e., safety, injury risks and potential to stunt growth) [18]. These issues may explain why few school-based physical activity interventions have focused on the promotion of RT [19]. Among those that have, most have been time intensive [20] and focused on the use of specialized weight training equipment [21]. Whilst these interventions may have improved both physical [20, 21] and psychological [21] outcomes on a small scale, there is a need to test school-based RT interventions that are scalable.

The need for widespread rollout of successful physical activity interventions has been well documented [22–25]. However, few school-based physical activity interventions have progressed beyond pilot, efficacy or effectiveness phases to be delivered at-scale [22, 26]. Developing effective interventions is only the first step toward improving population health. Transferring and sustaining effective programs into real world settings (such as schools) is a complicated and long-term process [27]. Scaling-up is the term used to describe the process of expanding the reach of efficacious health interventions under real-world conditions into broader policy or practice [28]. The Reach, Effectiveness, Adoption, Implementation and Maintenance (RE-AIM) framework [29] has been used extensively to evaluate the scale-up of effective health promotion interventions. RE-AIM allows for the assessment of both internal and external validity, which can help determine the public health impact of an intervention [30].

The *Nutrition and Enjoyable Activity for Teen (NEAT) Girls* [31] and *Active Teen Leaders Avoiding Screen-time (ATLAS)* [32] were two gender-targeted school-based interventions for girls and boys, respectively. They were designed to prevent obesity and promote physical activity among secondary school students in New South Wales (NSW), Australia. Positive findings from these programs included improvements in body composition [31], muscular fitness [32], and RT skill competency [32], as well as reduced recreational screen time [32, 33]. Despite their effectiveness, both programs included a large number of intervention components and required high levels of support from the research team, limiting their scalability [34]. Following the completion of the NEAT and ATLAS trials, the programs were adapted using feedback from participants [32], as well as our key stakeholder (i.e., the NSW Department of Education [DoE]). The goal of this process was to optimize [35] the intervention, through reviewing the existing intervention features, and identifying essential and non-essential components. This resulted in the new, consolidated, ‘*Resistance Training for Teens’* (*RT for Teens)* program*.*

The multi-component *RT for Teens* intervention was designed to improve muscular fitness and provide adolescents with the knowledge, motivation, skills, and confidence to engage in RT. The scalability of the intervention was optimized [35] through the inclusion of the following features: i) partnership with the NSW DoE; ii) flexibility of delivery within PE, school sport, or a PE-based elective subject (known as Physical Activity and Sport Studies); iii) reduction of program duration to fit within one school term (i.e., 10-weeks); iv) teacher-led delivery of the program, including training and resource provision; iv) smartphone application (app) to support program delivery; and v) greater focus on bodyweight exercises without the need for equipment or access to a gym.

*RT for Teens* was initially evaluated via a cluster randomized controlled trial (RCT) [36]. At the primary endpoint of 6-months, we observed significant improvements in adolescents’ muscular fitness, RT skill competency, and RT self-efficacy [36]. Improvements in muscular fitness and RT skill competency were also maintained at long-term follow-up (i.e., 12-months) [36]. Notably, students who were classified as overweight/obese had greater improvements in outcomes including upper body muscular fitness, RT skill competency, and motivation for RT [36]. The *RT for Teens* program followed a comprehensive pathway to scale-up, flowing through all four stages described by Indig and colleagues [37]. These four stages included: i) theory driven program development (i.e., guidance by Social Cognitive Theory and Self-Determination Theory); ii) testing for efficacy (i.e., original NEAT Girls [31] and ATLAS [32] programs); iii) testing for replicability/effectiveness (*RT for Teens* cluster RCT [36, 38]); and iv) the *RT for Teens* dissemination trial (i.e., the present study). Program development and rationale [39–41], and findings from the efficacy [32, 33] and effectiveness/replicability stages [36, 38] have been published previously. The aim of the current study was to evaluate the reach, effectiveness, adoption, implementation and maintenance of the *RT for Teens* program.

## Methods

### Study design

A type 2 hybrid effectiveness-implementation trial design [42] was used to assess both intervention outcomes and implementation strategies. Student- (reach, effectiveness), teacher- (adoption, implementation) and school-level (adoption, implementation, maintenance) data were examined [29]. The operationalisation of RE-AIM dimensions, RE-AIM data sources, outcome descriptions, and analytical methods are presented in Table 1. The RE-AIM framework [29] was chosen as the tool for evaluating the *RT for Teens* study as it balances internal and external validity, with the versatility to be used across all stages of research (from pilot to dissemination). Additionally, RE-AIM includes dimensions related to both outcome assessment and implementation quality [29], which are essential components when conducting a type 2 hybrid effectiveness-implementation trial [42]. Mixed methods were utilized, to maximize available data for the evaluation [43]. Ethics approval was obtained from the human research ethics committees of the University of Newcastle, Australia (H-2014–0312) and NSW DoE (SERAP: 2012121). Written informed consent was obtained from all school Principals and teachers. Study participants and their parents/caregivers were provided with information statements and opt-out consent was applied. The design and methods have been reported in detail previously [40]. *RT for Teens* was evaluated in two phases. The first phase (i.e., cluster RCT) took place in mid-2015 (ACTRN126150003605167). The second phase, (i.e., state-wide dissemination) commenced in late 2015 and was retrospectively registered (ACTRN12621000352808).

**Table 1:** RE-AIM operationalization, data sources and outcome detail

	**Reach**	**Effectiveness**	**Adoption**	**Implementation**	**Maintenance**
Operationalization	The estimated number and representativeness of students who may have been exposed to the program.	The impact of the program on students’ fitness, physical activity behaviors psychological constructs.	The number and representativeness of schools with at least one teacher trained (via the professional learning workshop) to deliver *RT for Teens.*	The extent to which the program was delivered as intended.	The extent to which the program was sustained in schools.
Data sources	i) *RT for Teens* teacher workshop enrollment data (to estimate student numbers). ii) School student enrollment data (from MySchool^a^ website; (https://www.myschool.edu.au) to evaluate dissemination cohort student characteristics.	i) Fitness tests and survey completion in a sub-sample of students (*n* = 750; from varied year levels) pre- (week 1) and post-program (week 10). Note: there was no control group A sub-sample of schools (*n* = 17) were selected through teacher interest following workshop completion. These teachers were asked to deliver the program for 10-weeks and were also asked to facilitate one lesson observation during the 10-week period (see *Implementation* for observation data).	i) *RT for Teens* teacher workshop enrollment data. ii) School data (from MySchool^a^ website) to evaluate dissemination and effectiveness sub-sample school characteristics. iii) Teacher demographics questionnaire (*n* = 429) Teacher questionnaire sent to all teachers prior to workshop attendance, with *n* = 429 (90%) completing it. iv) *RT for Teens* app.	i) *RT for Teens* session observations. ii) *RT for Teens* app usage Session observations were conducted at schools involved in the effectiveness sub-sample (*n* = 17; see *Effectiveness* for sub-sample information) to measure program implementation. One observation per class (*n* = 22 classes from the 17 schools) was conducted at the approximate mid-point of the 10-week (~ week 5) program period. Members of the research team, all of whom held a tertiary PE teaching qualification, and had been involved in the adaptation of the SAAFE principles to RT, conducted the lesson observations using a structured observation checklist. This measure has been used extensively in past school-based studies [36, 44], however it has not been validated. Number of workouts and fitness tests conducted using the app were also assessed to evaluate implementation of this program resource.	i) *RT for Teens* workshop enrollment data.
Outcome detail/s	Using teacher enrollment data (*n* = 468) from the workshops, we assumed that each teacher delivered the program to at least one class of 23 students (the mean class size in grades 7–10 in NSW). The characteristics of students enrolled in schools with at least one trained teacher (dissemination cohort) were collected from the MySchool^a^ database. Characteristics included: gender distribution, SES, Indigenous status, and language background other than English These characteristics were also collected and reported separately for schools involved in the effectiveness sub-sample (see *Effectiveness* for sub-sample information).	Sub-sample student-level data were collected pre- (week 1) and post-program (week 10). These assessments included fitness testing and survey completion. Fitness testing was completed by the research team, or by the classroom teacher who recorded results for the research team. Surveys were provided to schools as an online link, or as printed hard copies (only one school requested this due to internet unavailability). *Measures* Muscular fitness was assessed using the 90° push-up test (upper body muscular endurance) [45] and the standing long jump test (lower body power) [46]. Perceived fitness was reported using the IFIS, a 5-item instrument reporting perceptions on general fitness, and then separately for individual fitness components: CRF, muscular strength, speed/agility and flexibility [47]. RT self-efficacy was evaluated using a 5-item scale developed for use with adolescents [48]. Motivation to participate in RT was assessed using an adapted version of the BREQ-2 [49]. The intrinsic and identified subscales from the BREQ-2 [49] were utilized to evaluate autonomous motivation for RT using a 5-item scale. Participants self-reported their total physical activity and participation in muscle-strengthening physical activity using validated measures [50, 51].	Characteristics of schools (dissemination and effectiveness sub-sample) were collected via the MySchool^a^ database, and included selective status, sector, location, type, and Index of Community Socio-Educational Advantage based on school locality (providing a measure of schools’ area-level SES) Characteristics of teachers were also collected via survey prior to workshop attendance, including: age, sex, years of teaching experience, area of teaching specialty, other qualifications related to health-related fitness, and other recent professional development related to health and PE Adoption of the *RT for Teens* smartphone app was also operationalized, at the teacher and school level.	During these observations, researchers collected data on: i) fidelity and ii) adherence to SAAFE teaching principles [52] *Fidelity* Measured as the compliance with the proposed physical activity session structure (see Supplementary Table 1 and Supplementary Fig. 1a). This structure recommended 10 components to include within the session. During the observation, researchers assessed what components were being delivered to students during sessions, as well as whether teachers utilized any *RT for Teens* resources (i.e., circuit cards, smartphone app) *Adherence to SAAFE principles* The SAAFE principles, and strategies aligned with each principle were explained to teachers during the *RT for Teens* Professional Learning workshop. Adherence to the SAAFE principles was determined using a 16-item checklist, with items recorded on a 5-point scale (i.e., 1 = Not at all true to 5 = Very true), with a value assigned to each of the 16 specific strategies covered during teacher training (see Supplementary Fig. 1b). Based on these scores, a percentage was calculated by summing the mean for each of the strategies and dividing by the maximum possible score, for each SAAFE principle. Means for each strategy were also calculated.	Long-term follow-up of individual-level data were not collected during the dissemination trial. Workshop enrollment data were used to determine potential institutionalization of *RT for Teens*, where schools sent a new teacher/s, to an additional workshop after initial training.
Analysis	Descriptive statistics (mean, standard deviations, range) were utilized to report these data.	Linear mixed models were used to analyze outcomes using IBM SPSS Statistics for Windows, Version 20.0 (2010 SPSS Inc., IBM Company Armonk, NY), with significance set at *p* < 0.05. Models assessed the impact of time on the reported variable. Mixed models are consistent with the intention-to-treat principle, assuming data are missing at random.	Descriptive statistics (mean, standard deviations, range) were utilized to report these data.	Descriptive statistics (mean, standard deviations, range) were utilized to report these data.	Descriptive statistics (mean, standard deviations, range) were utilized to report these data.

*Abbreviations: BREQ-2* Behavioral Regulations in Exercise Questionnaire-2, *CRF* Cardiorespiratory fitness, *IFIS* International Fitness Scale, *NSW* New South Wales, *PE* Physical education, *RT* Resistance training, *SAAFE* Supportive, Active, Autonomous, Fair, and Enjoyable, *SES* Socio-economic status

**Fig. 1:**
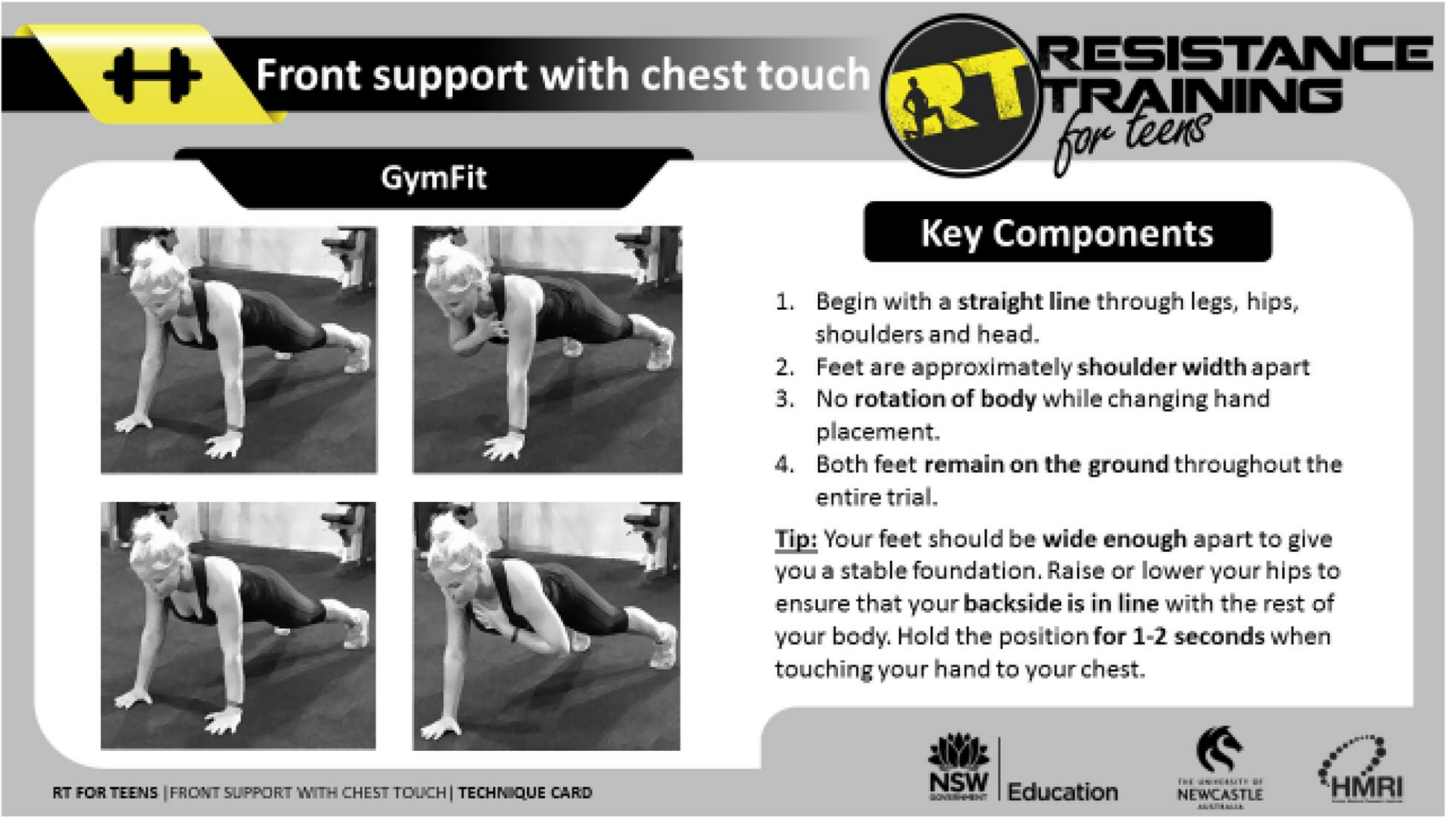
Circuit card example

**Fig. 2:**
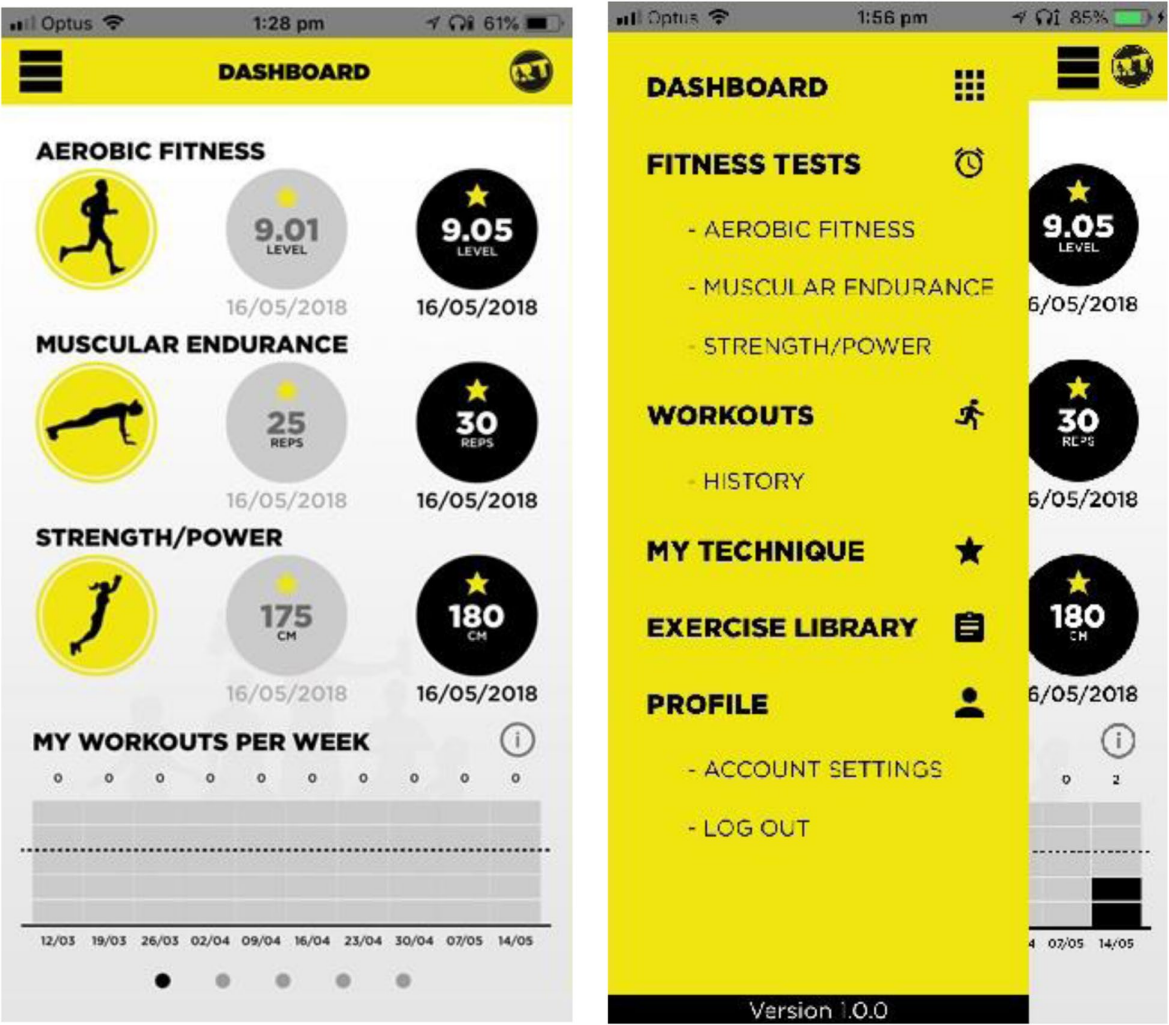
Smartphone app home screen and dashboard menu

All government and non-government secondary schools in NSW were eligible to participate in the dissemination phase. Dissemination has an inherent goal of adoption [53], whereby strategies are used to promote an intervention to the target population [54, 55]. Dissemination strategies were aimed at secondary school teachers as these were the initial target audience for the *RT for Teens* program. One strategy was the training of teachers to deliver the program, during a professional learning workshop delivered by the research team. This workshop was an accredited one-day training opportunity (i.e., teachers gained 5-h towards industry-mandated annual professional learning requirements), for teachers of any specialization. The workshop included practical and theoretical components designed to provide teachers with the knowledge, skills and competence to deliver the *RT for Teens* program. The workshop was adapted from the original RCT workshop to include the following content: (1) Program rationale including importance of muscular fitness, secular trends and RT guidelines, (2) Findings from the *RT for Teens* RCT, (3) Introduction to the *RT for Teens* program components, and (4) Motivating students using the SAAFE teaching principles. The final section did not highlight the physical limitations of specific groups (e.g., students with overweight or obesity). Instead it focused on delivering the *RT for Teens* program using autonomy supportive teaching practices. For example, students could choose to do push-ups on their knees or toes depending upon their perceived level of muscular fitness. Similarly, students were discouraged from comparing themselves to their peers, with the focus on self-improvement rather than competition. Teacher professional learning workshops (N = 30) were delivered from August 2015 until December 2019, with final data for the evaluation collected in October 2020. As an adjunct to the training, and to support program delivery, teachers were also provided with resources, including circuit cards (Fig. 1), access to a purpose-built smartphone app (Fig. 2 and Supplementary Fig. 2), and electronic copies of all workshop content.

Although teachers were the target audience of the workshop, it is important to note that teachers in NSW schools require approval from their head of department and school executive (i.e., principal or deputy principal) to attend professional learning workshops. As such, the decision to ‘adopt’ the *RT for Teens* program required support from the teachers, their head of department and the school executive. However, we acknowledge that teachers’ attendance at a professional learning workshop does not guarantee that they will successfully implement a program upon returning to school. Regarding implementation, schools were given considerable flexibility in how and when the program could be delivered. For example, it could be delivered as a school sport option, which did not require approval from the head of department. Alternatively, the program could be embedded within PDHPE curricula, which required head of department approval. Finally, we considered the sending of additional teachers to an *RT for Teens* workshop as an appropriate measure of maintenance because this demonstrates that schools were engaged with the program and wanted to “upskill” additional staff members. Similar to adoption, the decision to send additional teachers to another *RT for Teens* workshop required support from the teachers themselves, their head of department and the school executive.

## Results

### Reach

Based on the attendance of 468 classroom teachers at the *RT for Teens* professional learning workshop from 2015–2019, we conservatively estimate that the program reached ~ 10,000 students. This estimate is based on the assumption that teachers who attended training delivered the program to at least one class of 23 students (representing the average class size in NSW government secondary schools) [56]. Characteristics of students enrolled at schools with at least one trained teacher (dissemination cohort) are presented in Table 2. Just over half of students were male, with almost 30% from a language background other than English. Ten percent of students were of Indigenous heritage, and close to 40% from low socio-economic backgrounds. For comparison, characteristics of students enrolled at schools within the ‘effectiveness’ sub-sample are also presented in Table 2. Student characteristics were like that of the entire *RT for Teens* dissemination cohort, except schools within the effectiveness sub-sample had slightly larger enrollment numbers (~ 150 more students). Data available from NSW DoE specifies that 49% of school students are female [57]. This proportion is comparable to the percentage of females within *RT for Teens* dissemination cohort, effectiveness sub-sample school cohort, and effectiveness sub-sample student cohort (all ~ 48%). NSW data specifies that 6% of students identify as Aboriginal or Torres Strait Islander [57]. Data from the three *RT for Teens* groups indicates a slightly higher mean percentage of Indigenous students across these cohorts. Across the dissemination, effectiveness sub-sample school, and effectiveness sub-sample student cohorts, 10%, 8% and 7% reported Indigenous heritage, respectively.

**Table 2:** Characteristics of students enrolled in dissemination and effectiveness sub-sample schools

**Characteristics**	**Students from schools in the dissemination** **(*n* = 249)**	**Students from schools in the effectiveness sub-sample** **(*n* = 17)**
**Student enrollments**		
Total enrollments (N)	189,134	15,532
Female enrollments, n (%)	90,856 (48)	7,414 (48)
Male enrollments, n (%)	98,289 (52)	8,118 (52)
Enrollments per school, mean (SD)	760 (373)	914 (300)
Female enrollments per school, mean (SD)	365 (228)	436 (286)
Male enrollments per school, mean (SD)	395 (234)	477 (221)
**Language background other than English**^**a**^		
Range, %	0–100	2–93
Mean (SD), %	29 (31)	38 (33)
**Aboriginal or Torres Strait Islander decent**		
Range, %	0–81	0–35
Mean (SD), %	10 (11)	8 (10)
**Socio-economic status, mean (SD) %; range**^**b**^		
Low^c^	38.1 (22.1); 0–100	32.1 (24.1); 1–72
Medium^d^	46.0 (12.0); 0–74	48.1 (13.5); 26–64
High^e^	15.9 (18.2); 0–84	19.7 (20.3); 1–71

^a^ One school did not report the language background of its students

^b^ Data was unavailable for five schools

^c^ Percentage of students positioned in the lowest socio-educational advantage quartiles

^d^ Percentage of students positioned in the two middle socio-educational advantage quartiles

^e^ Percentage of students positioned in the highest socio-educational advantage quartiles

### Effectiveness

A total of 750 students from 17 schools located in a major city and inner regional areas provided effectiveness data. The characteristics of students in this sub-sample are presented in Table 3. Detailed findings for student-level outcomes are presented in Table 4. From this sample, statistically significant improvements from pre- (week 1) to post-program (week 10) were found for perceived cardiorespiratory fitness (CRF) (0.13 units, 95% CI: 0.07 to 0.19]), perceived flexibility (0.13 units, 95% CI: 0.05 to 0.21), participation in muscle-strengthening physical activities (0.70 days/week, 95% CI: 0.47 to 0.93), and RT self-efficacy (0.09 units, 95% CI: 0.03 to 0.14). In addition to questionnaire data, fitness measures were completed by 11 of the 17 schools. Statistically significant changes from pre- to post-program were found for push-ups (3.2 repetitions, 95% CI: 1.8 to 4.6) and the standing long jump (5.4 cm, 95% CI: 0.9 to 9.9).

**Table 3:** Baseline characteristics of students within the effectiveness sub-sample

**Characteristics**	**Effectiveness sub-sample (n = 750)**
**Age,**	
Mean (SD), y	14.4 (1.0)
Range, y	12–17
**Female participants, n (%)**^**a**^	357 (47.6)
**Born in Australia, n (%)**^**b**^	641 (85.5)
**English spoken at home, n (%)**^**b**^	644 (85.9)
**Cultural background, n (%)**^**b**^	
Australian	414 (55.2)
European	83 (11.1)
African	9 (1.2)
Asian	89 (11.9)
Middle Eastern	28 (3.7)
Other	125 (16.7)
**Aboriginal or Torres Strait Islander decent, n (%)**^**c**^	55 (7.3)
**Socioeconomic status, n (%)**^**d**^	
Low	147 (19.6)
Medium	268 (35.7)
High	329 (43.9)

^a^ Five students did not report their sex

^b^ Two students did not report their country of origin, language spoken at home, or cultural background

^c^ Five students did not report whether or not they were of Aboriginal or Torres Strait Islander decent

^d^ Socioeconomic status determined by population tertile using Socio-Economic Indexes For Areas of relative socioeconomic disadvantage based on residential postcode; six participants did not provide their residential postcode

**Table 4:** Analysis of outcomes

	**Baseline,**	**n**	**10-week,**	**n**	**Adjusted difference in change,** **Mean (95% CI)**^**a**^	**Time**
	**Mean (95% CI)**		**Mean (95% CI)**			***p***
**Fitness tests**						
Push-ups (reps)	15.18 (11.99,18.37)	435	18.39 (15.20,21.58)	422	3.21 (1.81,4.61)	** < 0.001**
Standing long jump (cm)	170.57 (162.27,178.88)	440	176.00 (167.69,184.30)	431	5.42 (0.93,9.92)	**0.023**
**Perceived fitness**^**b**^						
Perceived general fitness (units)	3.73 (3.58,3.90)	676	3.75 (3.60,3.90)	572	0.02 (-0.05,0.09)	0.527
Perceived CRF (units)	3.43 (3.36,3.49)	673	3.55 (3.48,3.62)	573	0.13 (0.07,0.19)	** < 0.001**
Perceived MF (units)	3.49 (3.38,3.60)	674	3.53 (3.42,3.64)	576	0.04 (-0.03,0.12)	0.225
Perceived speed/agility (units)	3.61 (3.54,3.67)	676	3.63 (3.56,3.70)	574	0.02 (-0.03,0.08)	0.392
Perceived flexibility (units)	3.07 (2.96,3.18)	668	3.20 (3.09,3.30)	574	0.13 (0.05,0.21)	** < 0.05**
**Self-reported participation in PA**						
Total PA (days/week)	3.71 (3.27,4.14)	710	3.93 (3.49,4.37)	566	0.23 (-0.90,0.55)	0.146
Strength-related PA (days/week)	2.08 (1.79,2.38)	659	2.79 (2.49,3.09)	567	0.70 (0.47,0.93)	** < 0.001**
**Motivation, self-efficacy and well-being**						
PA autonomous motivation (units)^c^	3.96 (3.91,4.03)	672	3.92 (3.85,3.98)	561	-0.05 (-0.10,0.01)	0.056
Motivation for RT (units)^c^	3.47 (3.34,3.59)	668	3.48 (3.35,3.62)	567	0.02 (-0.10,0.14)	0.743
RT self-efficacy (units)^d^	3.82 (3.71,3.93)	677	3.91 (3.79,4.02)	573	0.09 (0.03,0.14)	** < 0.05**

Abbreviations: *CI* Confidence intervals, *CRF* Cardiorespiratory fitness, *MF* Muscular fitness, *PA* Physical activity, *RT* Resistance training

^a^ Within group change over time from mixed model that included baseline and follow-up, and school as a random intercept

^b^ Perceived fitness scale: Participants report perceptions of their ‘general fitness’ and four other specific fitness components on a five-point scale, ranging from *Very poor* (1) to *Very good* (5)

^c^ Autonomous motivation for PA and Motivation for RT evaluated using a five-point scale, ranging from *Not true for me* (1) to *Very true for me* (5)

^d^ RT self-efficacy evaluated using a five-point scale, ranging from *Strongly disagree* (1) to *Strongly Agree* (5)

### Adoption

Between August 2015 and November 2019, 30 workshops were delivered, with 468 teachers from 249 schools in attendance. The characteristics of these schools are presented in Table 5. Most schools included grades 7–12 (traditional secondary school format), however some were split junior (grades 7–9 or 7–10) or senior (grades 10–12 or 11–12) campuses. Three of the adopting schools had atypical grade ranges (i.e., grades 3–12 and 5–8), as well as one primary (elementary; kindergarten to grade 6) school. Forty-six schools were fully or partially selective/specialist, and over 90% were Government funded schools. The 213 Government secondary schools that adopted *RT for Teens* represent almost half of all Government secondary schools in NSW [58]. Almost 70% of schools were located in major cities, whilst close to a quarter were from inner regional areas. The remaining 20% were in outer regional and remote areas. Participating schools’ mean ISCEA percentile was below the median (40%), ranging from the 2^nd^ to 99^th^ percentile. In addition to teachers from the aforementioned schools, eight employees from the NSW DoE School Sport Unit and the NSW Health Population Health research team attended workshops. This brings the total number of trained individuals to 476. Of these individuals, 429 (90%) completed a survey prior to attendance at the workshop to collect baseline demographic characteristics. Baseline characteristics of workshop attendees are presented in Supplementary Table 2. The majority of teachers were male, aged 26–30, and trained as a specialist PE teacher. Over half of the teachers did not have an additional qualification associated with fitness instruction, and average teaching experience was almost 12 years. Regarding adoption of the smartphone app, 144 of the 476 trained individuals created an app account (30%). At the school-level, 93/249 (37%) had at least one registered user (teacher and/or student). The number of users per school ranged from one to 335, with an average of 17 users per school.

**Table 5:** Dissemination school characteristics

**Characteristics**	**Dissemination Schools**
**School year range, n (%)**^**a**^	
7–12	203 (81.5)
K-12	24 (9.6)
7–10	10 (4.0)
11–12	4 (1.6)
10–12	3 (1.2)
5–8	2 (0.8)
7–9	1 (0.4)
3–12	1 (0.4)
K-6	1 (0.1)
**School sector, n (%)**	
Government	235 (94.4)
Non-Government	14 (5.6)
**Government secondary schools**	
Total, n (%)	213 (85.5)
**Selective/specialist schools, n (%)**^**c**^	46 (18.4)
Academic	19 (7.6)
Sporting	6 (2.4)
Special Education	6 (2.4)
Behavioral	5 (2.0)
Performing Arts	4 (1.6)
Intensive English	3 (1.2)
Agricultural	2 (0.8)
**School location, n (%)**^**d**^	
Major Cities	173 (69.5)
Inner Regional	56 (22.5)
Outer Regional	19 (7.6)
Remote	1 (0.4)
**Index of Community Socio-Educational Advantage (ISCEA)**^**e**^	
Percentile^f^	0.2 (28.4)
Range	2.0 – 99.0

^a^ Year ranges of schools that had at least one *RT for Teens* trained teacher

^b^ Government schools including students only from grades 7–12, including junior (7–9, 7–10) and senior (10–12, 11–12); for calculation of proportion against total NSW Government secondary schools

^c^ One school did not report the selective criteria

^d^ Remoteness classified by the Australian Bureau of Statistics, on the basis of a measure of relative access to services

^e^ This score is derived from a number of variables including parental school and non-school education and occupation, the school’s geographical location and proportion of Indigenous students; 1000 is average

^f^ The percentile of the school's ICSEA value, possible range = 1 to 100

### Implementation

During the dissemination evaluation, 22 lesson observations were conducted at the 17 schools within the effectiveness sub-sample. The level of implementation varied considerably across schools and is presented in Table 6. Resources were utilized in the majority of lessons, including the app and/or circuit cards. All lesson components (see Supplementary Table 1 and Supplementary Fig. 1a) were implemented to some degree, with the GymFit (i.e., development of RT skills) and high intensity resistance training (HIRT) workout the most prevalent. The behavioral messages and BoxFit (i.e., boxing style high intensity workout) were the least used components. Close to 60% of the 22 observed lessons included at least five of the suggested session components. Adherence to the SAAFE teaching principles was high, with Supportive and Active the most evident in sessions. Data from the *RT for Teens* app showed that of the 93 schools using the app, 48 used the workout function and 48 completed fitness testing. Thirty-five of the schools used both functions. In total, 2,336 workouts and 3,113 fitness tests were completed via the app.

**Table 6:** Process evaluation summary

**Intervention fidelity**	
Use of resources, mean (%)	59
Warm-up	
Includes movement-based game (1), mean (%)	55
Includes dynamic stretching (2), mean (%)	41
GymFit (3), mean (%)	77
HIRT workout (4), mean (%)	64
BoxFit (5), mean (%)^a^	36
CoreFit (6), mean (%)^a^	55
GameFit (7), mean (%)^a^	41
Cool down	
Includes static stretching (8), mean (%)	50
Behavioral messages discussed (9), mean (%)	23
Skill components reinforced (10), mean (%)	77
Overall session score, mean (/10)^b^	5
Lessons including > 50% of session components, mean (%)	59
**Adherence to SAAFE teaching principles**	
Supportive, mean (%)^c^	78
*Teacher provides individual skill specific feedback, mean (SD*^d^	3.8 (0.7)
*Teacher provides feedback on student effort and involvement, mean (SD)*^d^	4.0 (0.5)
*Teacher promotes positive interactions between students, mean (SD*^d^	4.0 (0.4)
Active, mean (%)^c^	78
*Activities involve small-sided games and circuits, mean (SD)*^d^	3.8 (0.9)
*Teacher monitors students’ activity levels (visually or using pedometers), mean (SD)*^d^	4.0 (0.4)
*Equipment is plentiful, mean (SD)*^d^	3.9 (0.8)
*Efficient transitions between activities, mean (SD*^d^	4.0 (0.8)
Autonomous, mean (%)^c^	64
*Teacher reinforces the relevance of the activities, mean (SD)*^d^	3.0 (0.8)
*Students are given choices about the tasks and activities, mean (SD)*^d^	3.3 (1.2)
*Students are involved in the set-up and running of activities, mean (SD)*^d^	3.6 (1.0)
Fair, mean (%)^c^	72
*Teacher ensures that students are evenly matched in activities, mean (SD)*^d^	3.4 (0.9)
*Teacher acknowledges and rewards good sportsmanship, mean (SD)*^d^	3.8 (0.5)
*If necessary, teacher modifies activities to maximize opportunities for success, mean (SD)*^d^	3.6 (0.8)
Enjoyable, mean (%)^c^	74
*Session starts with an enjoyable activity, mean (SD)*^d^	3.6 (1.2)
*Session finishes with an enjoyable activity, mean (SD)*^d^	3.7 (1.0)
*Session involves a wide variety of activities, mean (SD)*^d^	3.8 (1.0)

*Note:*^a^ Reasoning for the lower use of these session components compared to others may be explained by students/teachers being given the option to choose one of the three for Activity 4 within the proposed session structure (see Supplementary Table 1 and Supplementary Fig. 1a)

^b^ Calculated as the sum of all numbered (#) items within the intervention fidelity section

^c^ Calculated using the sum of all scores for that SAAFE element, divided by the highest possible score (i.e., 15 for supportive, autonomous, fair, and enjoyable; 20 for active)

^d^ on a 5-point scale ranging from not at all true (1) to very true (5)

### Maintenance

Fifty-one of the 249 schools sent at least one additional teacher to a RT for Teens workshop (38 schools sent one additional teacher, 12 schools sent two additional teachers and one sent three additional teachers), demonstrating that these schools were engaged with the program and wanted to “upskill” additional staff members.

## Discussion

The objective of our study was to evaluate the reach, effectiveness, adoption, implementation and maintenance [26] of the *RT for Teens* program. Our conservative estimate suggests ~ 10,000 students were exposed to the program, representing substantial reach. Regarding effectiveness, the program improved muscular fitness, RT self-efficacy, perceived CRF and flexibility, and participation in muscle-strengthening physical activities in a sub-sample of students who completed the assessments. Adoption was high, with 468 teachers from 249 schools trained to deliver the program. These 249 schools include almost half of the Government secondary schools in NSW. Implementation varied considerably across schools, however, resources usage and adherence to the SAAFE principles was evident in most sessions. A fifth of schools sent one or more teachers to subsequent workshops for training, indicating potential program maintenance in schools. To our knowledge, this is the first study focusing on the state-wide dissemination of a school-based RT program.

Previous studies have utilized a variety of methods to calculate the ‘potential’ reach of school-based physical activity interventions [59]. Similar to the current study, two interventions [60, 61] utilized teacher workshop enrollments to estimate potential reach into the student population. Others have used school enrollment numbers [62, 63], and ordering of program materials [64] as the method of calculation. Given the difficulty in collecting student participation data from teachers and schools at-scale, teacher workshop enrollment data was utilized as the method to estimate reach into the student population in the current study. Based on a conservative estimate that each teacher delivered the program to one class of students at their school, we estimated reach to be 10,000 students (5% of total student population). While it is possible that some teachers did not deliver the *RT for Teens* program to any of their classes, others may have delivered the program to multiple classes over a number of years since receiving the training (which commenced in 2015). As such, our estimate is likely to be an underestimation of actual student reach. Findings from semi-structured interviews conducted with teachers [65] support this notion, as teachers identified a variety of delivery methods, including during school sport, compulsory PE, and elective PE classes.

Of note, the *RT for Teens* program was also included in the *Physical Activity 4 Everyone (PA4E1)* whole-school intervention (as the enhanced school sport component) [66]. At least one teacher from each of the 24 PA4E1 program schools received the *RT for Teens* training [67] (these teachers are included in the aforementioned total of 468 trained teachers) [66]. Schools were instructed to deliver *RT for Teens* for 10-weeks to at least one full grade level of students [67], with 83% of schools achieving this at 12-months. With the inclusion of these schools, and the full grade of participating students, further support is provided for the potential underestimation of aforementioned reach into the student population.

Within the reach domain, it is also important to assess the characteristics of participants, to describe the representativeness of the population and thus the generalizability of findings [29]. This is important during at-scale delivery of population health interventions. The proportion of females within *RT for Teens* dissemination cohort, effectiveness sub-sample school cohort, and effectiveness sub-sample student cohort (all ~ 48%) was comparable to the 49% of female students reported across the NSW DoE [57]. Additionally, data from the three *RT for Teens* groups indicates a slightly higher mean percentage of Indigenous students across the dissemination, effectiveness sub-sample school, and effectiveness sub-sample student cohorts. These cohorts reported 10%, 8% and 7% students of Indigenous heritage, respectively, compared to 6% of NSW students identify as being of Aboriginal or Torres Strait Islander descent [57]. As such, these findings provide a positive insight into the availability of the *RT for Teens* program in schools with representative female and Indigenous student cohorts, however assumptions are preliminary, and limited due to available data.

There is considerable variability in the reporting of effectiveness data in programs delivered at-scale, with the majority reporting effectiveness from a prior study [59]. In the Action Schools! BC trial, efficacy was determined prior to scale-up and alluded to in future publications [61, 62]. Studies carried out in the school setting [68] have also used the educators’ perceptions of effectiveness (i.e., teachers’ perceived impact on student outcomes). The collection of valid outcome measures at-scale is challenging and as such, mixed methods are encouraged [69]. In addition to the quantitative data reported, semi-structured interviews aligned with RE-AIM were conducted with teachers to assess their perceptions of program impact. Findings from these interviews have been published previously [65]. Briefly, teachers reported high levels of student enjoyment, engagement, and motivation during the program [65]. The collection of student-level effectiveness data during the dissemination phase, via fitness tests and surveys [69], was an attempt to strengthen and support interview findings. Despite the lack of a control group to ascertain causality, the effectiveness findings from the dissemination can be interpreted in conjunction with those from our previous RCT [36]. This method was used in a recent systematic review to determine the scale-up penalty that occurs when interventions progress from efficacy to effectiveness to dissemination [70]. Of note, improvements in muscular fitness were slightly larger in the current study when compared to results from our previous RCT [36]. Considering the decreased effect often seen in scaled-up interventions (~ 60% scale-up penalty) [71], the effects observed in our effectiveness sub-sample are promising. However, due to the teacher-led collection of data in a number of schools, findings should be interpreted with a degree of caution. Whilst teachers were provided with instructions for fitness testing, and have been shown to conduct valid and reliable fitness testing [72], their level of training and commitment to rigor was likely lower than that of the research team. Additionally, a number of schools did not provide complete fitness data, which may have impacted our results. While lack of time was the main barrier to fitness testing, it is also possible that teachers may have forgotten to conduct tests and/or report results.

We operationalized adoption as the number of schools with at least one teacher who had participated in the *RT for Teens* professional learning workshop. A total of 468 teachers from 249 schools, including 213 Government secondary schools, had at least one *RT for Teens* trained teacher. Similar school numbers have been seen in previous successful school-based programs delivered at-scale, including SPARK PE [73]. A recent review [17] highlighted that ‘social influences’, such as support (or lack of) from school boards, is one of the most highly cited facilitators/barriers to the implementation (and preceding adoption) of school-based physical activity programs and policies. This finding provides a potential explanation for the adoption of *RT for Teens* in almost half of NSW government secondary schools [58]. The *RT for Teens* workshop was provided as accredited professional learning in partnership with the NSW DoE, with teachers gaining hours towards their mandated training requirements. In addition to this social influence, the partnership with the NSW DoE also allowed for co-creation of the adapted program. The research team utilized feedback from the DoE when adapting *RT for Teens* from the previous *NEAT Girls* [31] and *ATLAS* [32] programs. Co-creation is a necessary step when designing for dissemination, to maximize the contextual appropriateness of a program for a setting [74]. It is important that the needs of the stakeholders are met, to maximize adoption potential.

There was noticeable variation in implementation across schools. Of note, the overall session score within the dissemination (5/10) was lower than observed within the RCT (7/10) [36]. Despite the variations in delivery, the majority of lessons still included many of the key *RT for Teens* program components. The necessary focus on muscular fitness [74] remained (see Supplementary Table 1 and Supplementary Fig. 1a), as GymFit and HIRT workouts were the most commonly included components. Interview findings supported this observation, as teachers noted the increased incorporation of RT activities into lessons [65]. Variations in delivery during dissemination are not necessarily seen as a downfall, but rather a testament to the inbuilt flexibility of the program [40]. It is likely that during the dissemination phase, rather than delivering all program components in each lesson, teachers selected parts that best suited their students and the available lesson time. It is important to note that variation in delivery may impact outcomes, as certain program components (and/or the amount of time committed) may have differential impacts on students’ RT skills and muscular fitness. Balancing the need for adaptability whilst maximizing program impact is a constant tension in dissemination research [74]. Nonetheless, the effectiveness data from our sub-sample suggests the adaptations that may have occurred did not have an adverse impact on program outcomes, though this would need to be evaluated more rigorously to be certain.

The *RT for Teens* app was also a useful intervention resource, including fitness testing and workout functions. The current version of the app was only released in mid-2018, created in response to the reported barriers experienced with the earlier web-based version of the app [65]. Whilst app data provide an objective measure of resource utilization, the reported number of workouts and fitness tests may not be a true indication of usage. Strategies discussed during the professional learning workshop included how to overcome barriers related to device usage and student access to smartphones. These included how to make hardcopies of workouts and printed pre-post student fitness tests. As such, teachers may have created an app account and produced these hard copy resources, which limits the ability to assess true utilization of the app. App usage may have also varied throughout program delivery as students became more familiar with RT exercises, therefore requiring less support from the app. This was evident during the *RT for Teens* RCT [38], as resource usage (including the app) declined during the second half of the intervention period (as noted during lesson observations).

Whilst there are a number of promising findings related to implementation, there is considerable room for improvement. Although our study was designed using an established scale-up evaluation framework (i.e., RE-AIM), we did not use an existing implementation framework (e.g., Consolidated Framework for Implementation Research) [34] or scale-up guide (e.g., PRACTical planning for Implementation and Scale-up) [75]. In addition, our study was designed before publication of the Standards for Reporting Implementation Studies (StaRI) statement [76]. In the time period since *RT for Teens* was designed and developed, there has been rapid increase in the publication of recommendations and guidelines for implementation research [77]. Researchers now have added support to assist in the design, conduct and reporting of implementation trials than was available when the *RT for Teens* dissemination study was conceived.

Quality implementation of programs is linked with improved outcomes [16], so it is plausible that improvements in fidelity, would likely lead to greater improvements in student outcomes. Whilst program flexibility is a strength of *RT for Teens,* the lack of support following program training was a limitation. Our key learning from this study is that standalone professional learning workshops are not sufficient to support the high-quality implementation of physical activity programs in schools. External support from change agents is needed to overcome commonly faced barriers and enhance program implementation. The failure to provide on-going support may explain why ‘voltage drop’ occurs when interventions progress from small-scale projects with high levels of researcher support to larger-scale effective and dissemination studies with minimal researcher support. For example, one of the implementation strategies included within a recent school-based policy was to provided teachers with ongoing support [78]. This was via face-to-face meetings and remote communication (email and phone), between support officers and in-school champions. Similarly, teachers delivering a program for senior students were provided with initial (professional learning) and ongoing (observation and feedback) support [79]. Findings from these evaluations indicated that external support likely contributed to improvements in teachers’ implementation of the policy [78, 80, 81].

For the purpose of this study, maintenance was operationalized at the setting-level. School-level maintenance, or the integration of the program into practice (often referred to as institutionalization [29]), indicates that the intervention and implementation practices have been sustained. Long-term maintenance is dependent on teachers continuing implementation after the research evaluation period [82, 83]. Teacher-level factors such as confidence and understanding, along with perceived benefit to students, are important factors in determining implementation and maintenance of a program [16]. The proportion of schools with teachers attending an additional workshop (after the first instance of teacher training) was utilized as a representation of program maintenance (i.e., institutionalization of the *RT for Teens* program). Following interest, and incorporation of *RT for Teens* into one teachers’ practice, there arose a need to train additional teachers to sustain program delivery. At the very least this is an indication of teachers’ satisfaction with the workshop, and their belief that it would be of value to other staff members at their school. Interview findings support this assumption [65], with teachers reporting they were likely to share program information and resources with staff once they returned to school. This included with individual teachers and/or entire faculties through in-school workshops [65]. Individual-level (i.e., student) maintenance of program effects was demonstrated in the RCT [36], with muscular fitness and RT self-efficacy maintained at 12-months, however was not evaluated during the dissemination phase.

### Strengths and limitations

Strengths of this study include the extensive involvement of the NSW DoE School Sport Unit. This partnership allowed for greater dissemination of the program throughout NSW secondary schools. The mixed methods of data collection, including interviews, also allowed for the utilization of a variety of data sources to evaluate dissemination. Rather than relying on single sources of information, the multiple methods allowed for a more in-depth exploration of program impact across RE-AIM domains. Evaluation of program effectiveness via the RCT, prior to at-scale dissemination, also contributed to the strength of this study. This data allowed for findings from the dissemination phase (where there was no control group) to be compared against outcome improvements from the rigorous RCT. Whilst this study had many strengths, it is not without limitations. Firstly, little implementation support was provided to teachers following the workshop, which may have hindered implementation efforts. Second, this lack of support/contact with schools also presented as a barrier to determine implementation quality and maintenance. Third, not all schools used the *RT for Teens* app. According to our usage data, participants completed 2,336 workouts and 3,116 fitness tests. These numbers are much lower than our estimated reach (i.e., ~ 10,000 students). Fourth, implementation findings were determined using data from a subset of participating teachers. Fifth, program acceptance data was not collected from students and teachers in the dissemination phase. However, findings from our RCT suggest that teachers were highly satisfied with the program (4.8/5) [38]. Students’ overall satisfaction with the program was not quite as high (3.8/5) [36]. Finally, evaluation of implementation quality and the impact of varied delivery methods on student-level outcomes was not explored.

## Conclusions

Given the need to implement effective physical activity programs at-scale [22–25], our dissemination study provides an important contribution to the field. *RT for Teens* is the first school-based RT program to be delivered at-scale. Our study provides an in-depth account of the journey of the program through scale-up and describes the impact of the program across RE-AIM dimensions. The *RT for Teens* program had large potential reach and high levels of adoption, however, implementation varied considerably across schools. External support from change agents may be needed to overcome barriers and optimize intervention implementation in schools, including the documentation of adaptations that may have been made to increase implementation success. Future studies, guided by accepted frameworks, recommendations and guidelines [34, 75–77] for implementation research are needed. These will further explore and evaluate the impact and cost-effectiveness of RT interventions in schools, with a greater focus on the impact of implementation support on implementation quality and program maintenance.

## Supplementary Information

**Additional file 1: Supplementary Table 1**. Proposed RT for Teens session structure.

**Additional file 2: Supplementary Table 2**. Characteristics of workshop attendees.

**Additional file 3**: The TIDieR (Template for Intervention Description and Replication) Checklist*.

**Additional file 4: Supplementary Figure 1a**. RT for Teens session observation checklist (page 1, session components). **Supplementary Figure 1b**. RT for Teens session observation checklist (page 2, SAAFE adherence).

**Additional file 5: Supplementary Figure 2**. RT for Teens exercise library examples (from App).

## Data Availability

Study data and materials are not available publicly, however may be available upon request to the lead investigators. All consenting participants were issued a unique identification number for confidentiality, and all data is stored securely as per ethical requirements.

## Abbreviations

ATLAS: Active Teen Leaders Avoiding Screen-time
BREQ-2: Behavioral Regulations in Exercise Questionnaire-2
CI: Confidence intervals
CRF: Cardiorespiratory fitness
DoE: Department of Education
HIRT: High intensity resistance training
IFIS: International Fitness Scale
NEAT: Nutrition and Enjoyable Activity for Teen
NSW: New South Wales
PA4E1: Physical Activity 4 Everyone
PE: Physical education
RCT: Randomized controlled trial
RE-AIM: Reach, effectiveness, adoption, implementation, maintenance
RT: Resistance training
SAAFE: Supportive, active, autonomous, fair and enjoyable
SES: Socio-economic status
US: United States
