# Correction to: Evaluating the reach, effectiveness, adoption, implementation and maintenance of the Resistance Training for Teens program

**DOI:** 10.1186/s12966-021-01229-1

**Published:** 2021-11-30

**Authors:** Sarah G. Kennedy, Jordan J. Smith, Paul A. Estabrooks, Nicole Nathan, Michael Noetel, Philip J. Morgan, Jo Salmon, Gessika C. Dos Santos, David R. Lubans

**Affiliations:** 1 Priority Research Centre for Physical Activity and Nutrition, School of Education, University of Newcastle, Callaghan, NSW Australia; 2 Department of Health Promotion, University of Nebraska Medical Center, Omaha, NE USA; 3 National Centre of Implementation Science, University of Newcastle, Callaghan, NSW Australia; 4 Hunter New England Population Health, Hunter New England Area Health Service, Newcastle, NSW Australia; 5 College of Health, Medicine and Wellbeing, The University of Newcastle, Newcastle, NSW Australia; 6 Hunter Medical Research Institute, New Lambton Heights, NSW Australia; 7 School of Health and Behavioural Sciences, Australian Catholic University, Sydney, NSW Australia; 8 Institute for Physical Activity and Nutrition (IPAN), Deakin University, Geelong, Australia; 9 Post-Graduate Program in Physical Education Associate UEM/UEM, State University of Londrina, Londrina, Brazil

**Correction to: Int J Behav Nutr Phys Act 18, 122 (2021).**

**https://doi.org/10.1186/s12966-021-01195-8**

Following the publication of the original article [1], the authors identified errors in the author name and affiliation.

The incorrect author name is: Mike Noetel.

The correct author name is: Michael Noetel.

The incorrect affiliation is: Institute for Positive Psychology and Education, Australian Catholic University, Sydney, NSW, Australia.

The correct affiliation is: School of Health and Behavioural Sciences, Australian Catholic University, Sydney, NSW, Australia.

The author group has been updated above and the original article [1] has been corrected.
