# Supplementary material for: Evaluating the reach, effectiveness, adoption, implementation and maintenance of the Resistance Training for Teens program
Source: Int J Behav Nutr Phys Act. 2021 Sep 8;18:122. doi: 10.1186/s12966-021-01195-8 (PMC8425054; doi:10.1186/s12966-021-01195-8)
Supplement: Supplementary file 1 — Additional file 1: Supplementary Table 1. Proposed RT for Teens session structure. [file 12966_2021_1195_MOESM1_ESM.docx]

**Supplementary Table 1**. Proposed *RT for Teens* session structure

| **Activity** | **Purpose** | **Explanation** | **Timing** |
| --- | --- | --- | --- |
| 1. Warm-up | - Safety | - General warm-up involving:   - Movement based games   - Dynamic stretching | 3-5 mins |
| 2. GymFit | - Develop RT movement skills | - Circuit or workout consisting of Gymstick and body weight exercises - Opportunities for student choice - Moderate intensity exercise - Emphasis on skill development and improving technique | 20-30 mins |
| 3. HIRT workout | - Improve muscular and cardiorespiratory fitness | - Short, high intensity workout - Intermittent work:rest ratio - Pre-designed or self-selected workout completed for 4-13 minutes - Performed in pairs. One partner works, the other reminds of exercises and motivates. After workout complete, they swap. | 8-26 mins |
| 4. Select from:  i) GameFit  ii) BoxFit  iii) CoreFit  iv) CardioFit | - Enjoyment - Student choice | - Students may decide to participate in a boxing/aerobic circuit (i.e., BoxFit/CardioFit), a Yoga/Pilates session (i.e., CoreFit), or play a modified game with fitness infusion (i.e., GameFit). - Teachers may facilitate multiple activities during this period | 20-30 mins |
| 5. Cool down | - Reinforce messages - Consolidate learning | - Static stretching and light activity - Discuss *RT for Teens* behavioral messages - Reinforce key skill components or concepts | 5 mins |
