# Supplementary material for: Evaluating the reach, effectiveness, adoption, implementation and maintenance of the Resistance Training for Teens program
Source: Int J Behav Nutr Phys Act. 2021 Sep 8;18:122. doi: 10.1186/s12966-021-01195-8 (PMC8425054; doi:10.1186/s12966-021-01195-8)
Supplement: Supplementary file 2 — Additional file 2: Supplementary Table 2. Characteristics of workshop attendees. [file 12966_2021_1195_MOESM2_ESM.docx]

**Supplementary Table 2.** Characteristics of workshop attendees

| **Characteristics** | **Total (n=429)** |
| --- | --- |
| **Age range, y, n (%)**  21-25  26-30  31-35  36-40  41-45  46-50  51+ | 37 (8.6)  113 (26.3)  71 (16.6)  67 (15.6)  59 (13.8)  31 (7.2)  51 (11.9) |
| **Female participants, n (%)** | 177 (41.3) |
| **Years of teaching experience, mean (SD)** | 11.7 (8.3) |
| **Area of teaching specialty, n (%)^a^**  PE  Other | 377 (87.9)  51 (11.9) |
| **Other qualifications related to fitness instruction, n (%)**  Yes  No | 186 (43.4)  235 (54.8) |

^a^ One teacher did not report their specialty

^b^ Eight teachers did not report whether or not they had additional qualifications.
