# Supplementary figures and images for: Evaluating the reach, effectiveness, adoption, implementation and maintenance of the Resistance Training for Teens program
Source: Int J Behav Nutr Phys Act. 2021 Sep 8;18:122. doi: 10.1186/s12966-021-01195-8 (PMC8425054; doi:10.1186/s12966-021-01195-8)

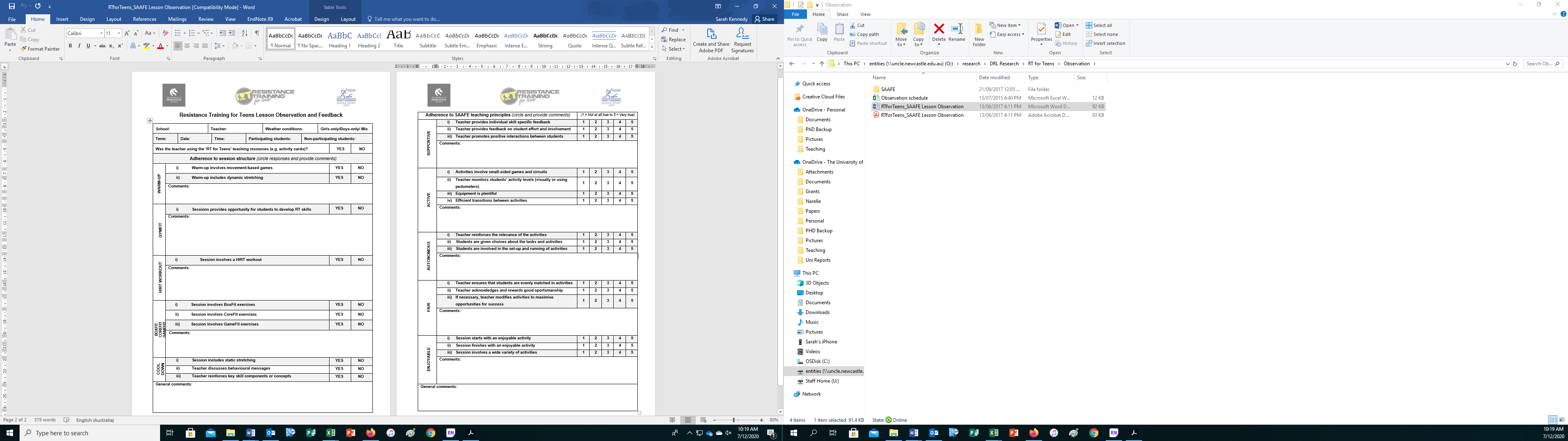


**Supplementary Figure 1a.** RT for Teens session observation checklist (page 1, session components)

Supplement: Supplementary file 4 — Additional file 4: Supplementary Figure 1a. RT for Teens session observation checklist (page 1, session components). Supplementary Figure 1b. RT for Teens session observation checklist (page 2, SAAFE adherence). [file 12966_2021_1195_MOESM4_ESM.docx]

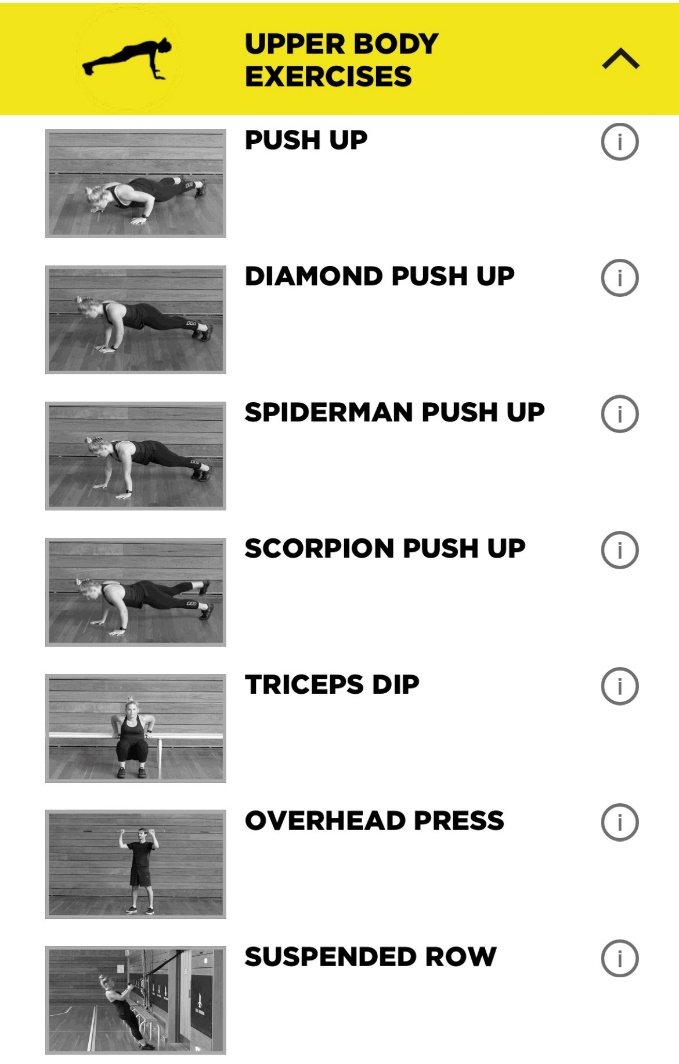

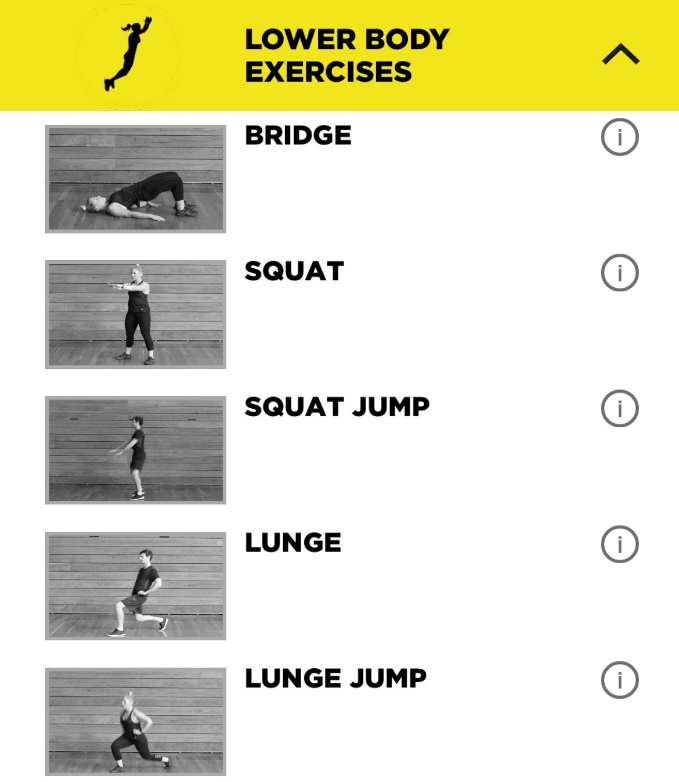

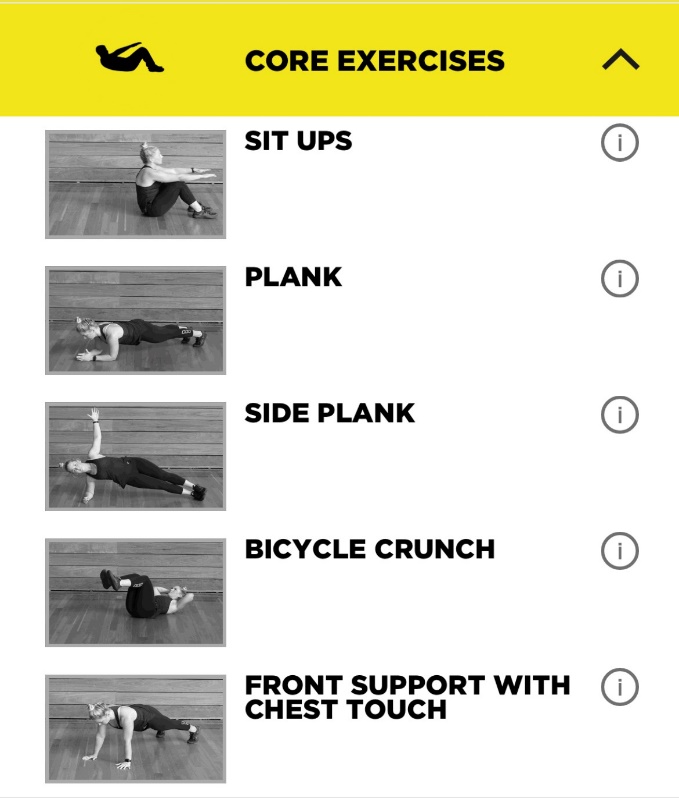


**Supplementary Figure 2.** RT for Teens exercise library examples (from App)

Supplement: Supplementary file 5 — Additional file 5: Supplementary Figure 2. RT for Teens exercise library examples (from App). [file 12966_2021_1195_MOESM5_ESM.docx]
